# Supplementary material for: Two Conserved Amino Acids Characterized in the Island Domain Are Essential for the Biological Functions of Brassinolide Receptors
Source: Int J Mol Sci. 2022 Sep 28;23(19):11454. doi: 10.3390/ijms231911454 (PMC9570414; doi:10.3390/ijms231911454)
Supplement: Supplementary file 1 [file ijms-23-11454-s001.zip › Supplementary materials.pdf]

Supplementary materials

Two conserved amino acids characterized in island domain are essential for biological functions of brassinolide receptors

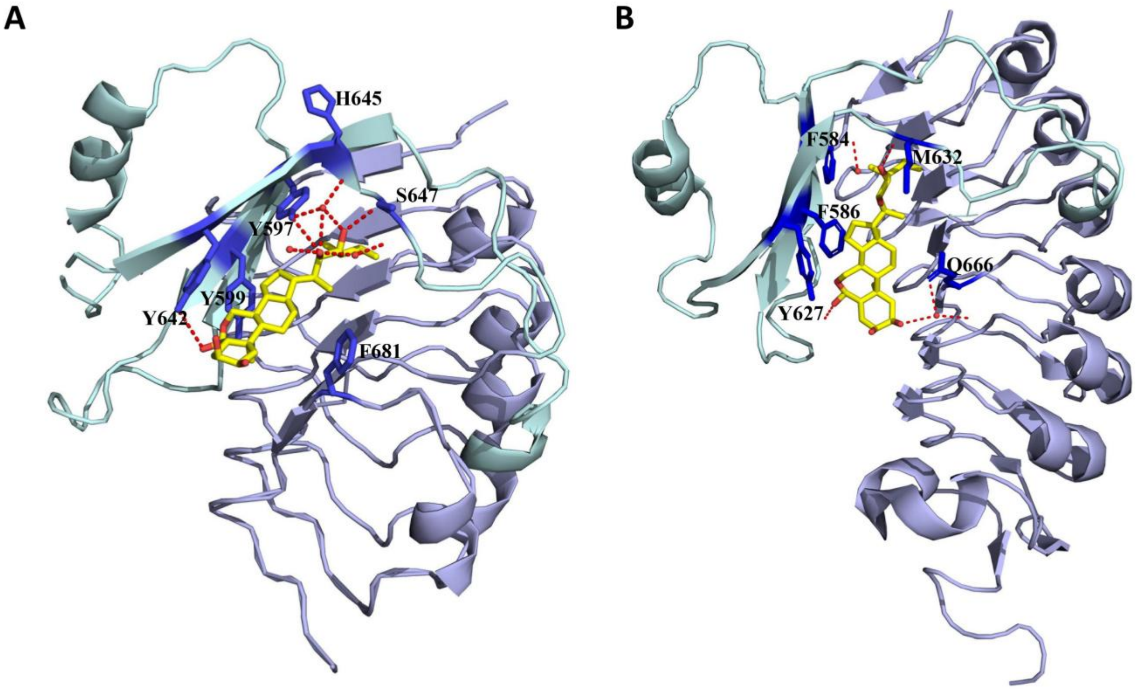

Supplementary Figure S1. Crystal structures comparison of BL binding pockets of BRI1 with BRL1. **(A, B)** Both the LRRs 18-25 of BRI1(3RGX) **(A)** and LRRs 18-24 of BRL1(4J0M) **(B)** are colored in light blue, and their ID domains are highlighted in pale cyan. The contacting residues (in blue) are presented with full side-chains. Polar interactions are shown with dotted lines, water molecules are represented by red spheres, and the BL is depicted in yellow.

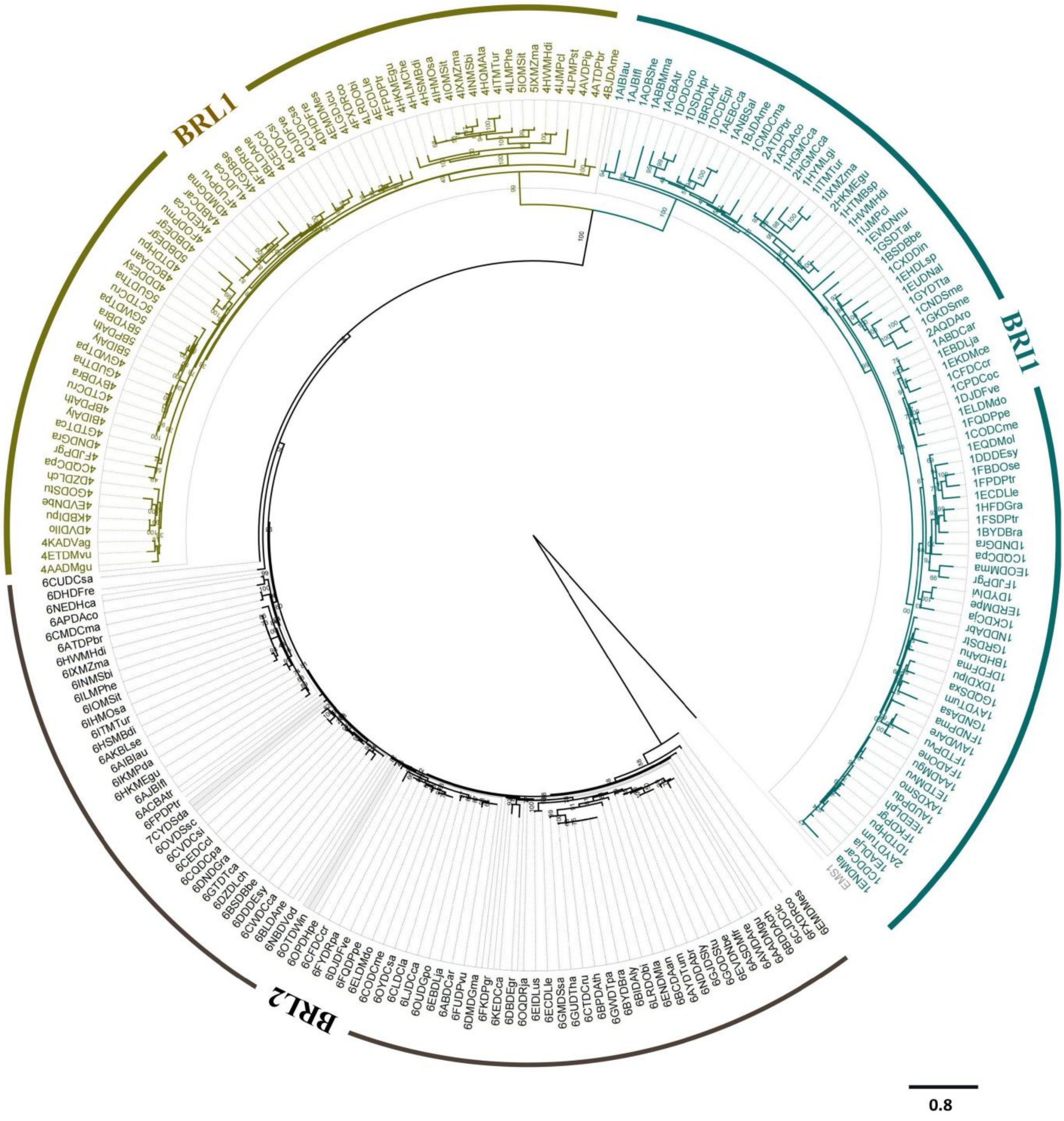

Supplementary Figure S2. Bayesian phylogeny of IDs from different species of angiosperm. All the IDs of BRI1 from different species are presented in cyan, the IDs of BRL1/3 are indicated in goldenrod, and the IDs of BRL2 are depicted in black. EMS1 serves as an outgroup. The confidence values shown beside each node are estimated using the SH-aLRT branch test with 1000 replicates. Scale bar refers to a phylogenetic distance of 0.8 amino acid replacements per site per unit of evolutionary time.

```
            1        10        20        30        40        50        60
1AADMgu   SGNI.AVGLL.TGKSYVYIKNDG.SQQCHGAGNLLEFGGIRQEQLNRI.STRHPCNFTRVYRGIT.QPTFNHNG
1ETDMvu   SGNI.AVGLL.TGKSYVYIKNDG.SKQCHGAGNLLEFGGIRAEQLNRI.STRHPCNFTRVYKGIT.QPTFNHNG
1AUDPdu   SGNI.AAALL.TGKSYVYIKNDG.SKQCHGAGNLLEFGGIRQEQLNRI.STRHPCNFTRVYRGIT.QPTFNHNG
2AYDTum   SGNI.ADALL.TGKSYVYIKNDG.SKECHGAGNLLEFGGIRQEQLNRI.STRHPCNFTRVYRGIT.QPTFNHNG
1FKDPgr   SGNI.AAAYL.TGKPYVYIKNDG.SKQCHGAGNLLEFGGIRQEQLDRI.STRHPCNFTRVYRGIT.QPTFNHNG
1EADLja   SGKI.ADALL.TGKSYVYIKNDG.SQECHGAGNLLEFGGIRQEQLDRI.STRHPCNFTRVYRGIT.QPTFNHNG
1DXDIpu   SGNI.AMALL.TGKQYVYIKNDG.SKECHGAGNLLEFGGITQERLDRI.STRHPCNFTRVYKGIT.QPTFNHNG
1AXDSmo   SGNI.AVGLL.IGKSYVYIKNDG.SKECHGAGNLLEFGGIRAEQLHRI.STRHPCNFTRVYKGIT.QPTFNHNG
1NDDAbr   SGNI.AVSLL.TGKQYVYIKNDG.SKECHGAGNLLEFGGIRQENLNRI.STRHPCNFTRVYRGIT.QPTFNHNG
1GRDStr   SGNI.ALGLL.TGKRYIYIKNDG.SKQCHGAGNLLEFGGIRLEQLDRV.STRHPCNFTRVYRGIT.QPTFNHNG
1GQDSxa   SGNI.ALALL.TGKRYLYIKNDG.SKECHGAGNLLEFGGIREEQLDRI.STRHPCNFTRVYKGVI.QPTFTRNG
1CDDCar   SGKI.DVALL.TGKSYVYIKNDG.SKQCHGAGNLLEFGGIRQEQLNRI.STRHPCNFTRVYKGIT.QPTFNHNG
1EEDLph   SGKI.AAAVL.TGKSYVYIKNDG.SKQCHGAGNLLEFGGIRQEQLNRI.STRHPCNFTRVYRGIT.EPTFNHNG
1DTDHpu   SGYI.AAAFL.TGKRYMYIKNDG.SKQCHGAGNLLEFGGIGQDQLNRI.STRHPCNFTRVYRGIT.QPTFNHNG
1DFDFma   SGNI.AVGLL.TGKRFIYIKNDG.SKQCHGAGNLLEFGGIRQDQLNRI.LTRHPCNFTRVYGGIT.QPTFNHNG
1AYDTum   SGKI.ALAVL.TGKRYVYIKNDG.SKQCHGAGNLLEFGGIRQEQLHRI.STRHPCNLTRVYPGMT.QPTFNHNG
1FNDPma   SGQI.AVSWL.TGKRYVYIKNDG.SRQCHGAGNLLEFGGIRPEQLVRI.STRNPCNFTRVYKGIT.QPTFENNG
1BHDAhu   AGRI.LLGLL.TAKQYIYIKNDG.SKQCHGAGNLLEFDGIRQEQLSRI.SSRHPCNFTRVYRGIV.APTFNHNG
1ABDCar   SGKI.AVNFI.SGKTYVYIKNDG.SKECHGAGNLLEFAGISQQQLNRI.STRNPCNFTRVYGGKL.QPTFNHNG
1EBDLja   SGKI.RVNFI.SGKTYVYIKNDG.SRECHGAGNLLEFAGISQQQLNRI.STRNPCNFTRVYGGKI.QPTFKNTG
1EKDMce   SGNI.AVNFI.SGKTYVYIKNDG.SKECHGAGNLLEFAGIRHSQLNRI.STMNPCNFTRVYDGKI.QPTFSNNG
1EQDMol   SGKV.AVNFI.AGKRYVYIKNDG.SKACHGAGNLLEFAGIRPEQLSRI.STRSPCNFTRVYGGHT.QPTFNNNG
1DNDGra   SGKI.AVNFI.AGKRYMYIKNDG.SRECHGSGNLLEFAGIRSEDLNRI.SDRNPCNFTRVYGGHT.QPTFNNNG
1CPDCoc   SGKI.ALNFI.TSKTYVYIKNDG.SKECHGAGNLLEFAGIRQEQLNRI.STRNPCNFTRVYRGSV.QPTFKHEG
1DJDFve   SGNI.AVNFV.ASKTYVYIKNDG.SKECHGAGNLLEFAGIRQEQLTRL.STRNPCNFTRVYRGIL.QPTFNHNG
1ELDMdo   SGNI.AVNFV.ASKTYVYIKNDG.SKECHGAGNLLEFAGIRTEQLNRI.STRNPCNFTRVYRGIL.QPTFNHNG
1FQDPpe   SGNI.AVNFI.VSKTYAYIKNDG.SKECHGAGNLLEFAGIRDEHLNRI.SARNPCNFTRVYRGMI.QPTFNHNG
1CODCme   SGNI.AVNFI.TGKSYAYIKNDG.SKQCHGAGNLLEFAGIRQEQVSRI.SSKSPCNFTRVYKGMT.QPTFNHNG
1CFDCcr   SGSI.AVKYL.SQKTYVYIKNDG.SKECHGAGNLLEFAGIDHEQLNRI.STRNPCNFTRVYEGKL.QPTFYHNG
1CQDCpa   SGKI.AANFI.AGKKYAYIKNDG.SRACHGAGNLLEFAGIREEQLNRI.SAWNPCNFTRVYRGLT.QPTFNNNG
1FSDPtr   SGKI.AANFI.AGKRYVYIKNDGSKKQCHGAGNLLEFQGIRPEQLTRL.SVRNPCNFTRVYGGHT.QLTFNNNG
1BYDBra   SGKI.AVNFI.AGKRYVYIKNDGMKKECHGAGNLLEFQGIRWEQLSRV.STRNPCNFTRVYKGHT.SPTFDNNG
1HFDGra   SGKI.AANFI.AGKRFAYIKNNG.SIMCHGAGNLLEFQGIRSEQLTRL.SARSPCNFTRVYGGHT.QPTFNNNG
1DYDIvi   AGNI.AVNLV.KGKKYAYIKADG.SHECHGAGNLLEFAGIRQEQLSRI.SARNSCNFTRVYGGKT.EPTFNHNG
1ERDMpe   AGNI.AVNLV.KGKRYAYIKADG.SEECHGAGNLLEFAGIRQAQLSRI.SVRNPCNFTRVYGGKT.EPTFNHNG
1FBDOse   SGNI.AVNFI.AGKRYAYLKNEK.SDRCHGAGNLLEFAGIRSEELDRI.SSRHPCNFTRVYGGHT.QPAFKNNG
1FPDPtr   SGSI.AVNFI.RGKRYVYLKNEK.SEQCHGEGDLLEFAGIRSEHLIRI.SSRHPCNFTRVYGDYT.QPTFNDNG
1ECDLle   SGNI.AVNFI.TGKRYVYLKNDK.SQNCHGTGNLLESQGIRPEKFFWI.ATRHPCTFTRVYGGHT.QPTFNNNG
1DDDEsy   SGNI.SPTSI.AGKRFAYLKNDG.SKQCHGAGNLLEFAGIRPEQLSRI.STRSPCNFTRVYNGHT.QPTFNYNG
1AWDAre   SGNI.AVALL.TGKRYVYIKNDGSSRQCHGAGNLLEFGGVRDDQLDRI.STRNPCN.GRVWRGIT.QPNFNHNG
1FADOne   SGNI.AVALL.TGKRFLYIRNDG.SKQCHGAGNLLEFGGIRDDQLDRI.LTRNPCN.GRVWRGTT.QPNFNHNG
1FTDPvu   SDYI.DVALL.TGKRYIYIKNNG.SKQCHGAGNLLEFGGVRDEEQDRI.TTRHSCSVGLVWRGIT.QPNFNHNG
1EODMma   SGGI.EVKIN.TDKRYAYIKNDG.SKACHGAGNLLEFAGVRPEQLNRI.STRNPCNFTRVYNGLL.KPTFKNNG
1FJDPgr   SGKI.TVKLL.NDKRYAYIKNDG.KKECHGAGNLLEFAGIRPEQLNRM.STRNPCNFTRVYKGVI.DPTFKNNG
1ENDMla   SGSI.AVAML.TGKSYRYIKNDG.SEQCHGAGNLLEFGGIREEQFTRI.LTRQPCNFTRVYGGPV.KPTFNQNG
1CKDCja   SGKI.GVGLVNTGKRYSYIKADG.SKECHGAGNLLEYAGIRPEQLNRI.STRSPCNFTRVYGGGI.QPTFNQNG
1ACBAtr   SGNI.AAGLV.TGKTYAYLRNDG.SSNCHGAGNLLEMAGIRTENLARV.PTRRSCNFTRIYLGKT.SYTFKNNG
1DODGro   SGKI.AVALI.AGKSYAYLKNDG.SSNFHGAGNLLEFAGIRQEGLTRI.PTWRQCNFTRIYIGKT.AYMFKNNG
1DSDHpr   SGKM.AVALI.AGKSYVYLKNDG.SNNCHGAGNLLEFSGIRQDGLTRV.PTRRQCNFTRIYIGKT.AYIFNKNG
1AIBIau   SGNI.AVGLV.TGKRYVYLRNDG.SSNCRGAGNLFEFAGIQMDQLNRV.PTLRSCNLSRIYRGIT.QYTFKNNG
1AOBShe   SGNI.AVGLV.SGKRYVYMRNDG.SIDCRGAGNLLEFSGIRPEQLNRV.PTRHSCNFTRIYQGNT.QYTFNNNG
1CMDCma   SGNI.AVGLI.TGKRYVYLKNDG.SPDCHGAGNLLEFAGIRPEGLNRV.PTRHSCNFTRVYKGNA.QYTFNNNG
1BJDAme   AGNI.AAGLV.TGKRYVYLKNDG.SADCHGAGSLLEFAGIRQDALNRV.PTRNSCNFTRIYMGNT.QYTFDNNG
1BRDAtr   SGKI.AVGLV.IGKRYVYLKNDG.SIGCHGAGNLLEFAGIRQDELNRV.PTRRSCNFTRVYMGNT.QYTFANNG
1APDAco   SGNI.AVGLV.TGKKYVYLKNDG.STGCRGAGNLLEFAGIRPDGLNRV.PTRRSCNFTRVYMGNT.QYTFDHNG
1HTMBsp   SGKI.AGGLV.TGKRYVYLKNDG.SKECRGAGNLLEFAGIRQDQLNRL.PSHRSCNFTRVYMGST.KYTFNNNG
2HKMEgu   SGNI.AVGLV.TGKRYVYLRNDG.SSECRGSGNLLEFAGIRPEELNRL.PSRRFCNFTRPYMGST.RYTFNNNG
2ATDPbr   AGQI.GVGWI.SGKRYVYLKNDG.SSECHGAGNLLEFSGIRPEGLDRV.PTRSSCNFTRIYMGNT.EYTFSNNG
1HYMLgi   SGKV.VGGLL.TGKRYVYLRNDE.NSQCHGTGSLLEFGGIRSDDLTRM.PSKRLCNFTRVYMGNT.QSTFNNNG
1HWMHdi   SGSI.AVSFV.TGKRYVYLKNDG.SKECRGAGNLLEFAGIRPEQLSRL.PS.HPCNFTRVYVGST.RYTFNKKG
1IJMPcl   SGNI.AVGFV.TGKRYVYLKNDG.SKECRGAGNLLEFAGIRPEQLSRL.PS.HPCHFTRVYMGST.RYTFNKKG
2HGMCca   SGKI.AVGLV.TGEPYVYLKNDG.NSGCRGTGDLLEFAGVRPEDLDRL.SSRQFCNFTRVYKGIT.QYTFNNNG
1DCDEpl   SGKI.AVGLI.TGKSFVYLKNDM.SIDCRGAGNLFEFAGIRQDGLNRV.PTWGSCNFTRAYKGLT.RYTFNNNG
1BSDBbe   SYKI.AVGLV.IGKTYAYLRNDG.SKECHGAGNLLEFSGIRQEQLDRV.PTRQSCNFTRVYRGNT.QPTFNHNG
1EUDNal   SGNM.PGGLV.LGKKFVYLKNDG.SKECHGAGNLLKFSGIRQNALDRI.PTRRSCNFTRIYKGNT.QPNFNHNG
1GYDTta   SGNM.AAGLV.VGKKYIYLRNQW.SKECHGTGNLLEFAGIREQALDRI.PTRDTCNFTQIYRGNTLQPNFNRNG
1CXDDin   SGII.AVGLV..GKRYAYVRNDG.SKECHGAGNLLEFSGIRDDQLSRI.PTK.SCNFTVVYRGKT.QPTFNHNG
1EHDLsp   SGKL.AVGLV.VGKKYVYLRNDG.SKECHGAGDLFEFSGIRETVLNQI.PSRQTCNFTRIYRGNT.LPTFQHNG
1ITMTur   SGKM.TVGLI.IGRPYVYLRNDELSSQCRGKGSLLEFSSIRSEDLGRM.PSKKLCNFTRMYMGST.EYTFNKNG
1IXMZma   SGKM.NVGLV.LGRPYVYLRNDELSSECHGKGSLLEFTSIRPEELSRM.PSKKLCNFTRVYMGST.EYTFNKNG
1GSDTar   SGKI.TVGLI.TGKRYVYLRNDG.SKECHGVGNLLEFAGMRQNSLNRV.PTRHSCNF.RAYPGIT.KYNFDHNG
1AEBCca   SGKI.AEGLV.TGKKFVYLKNNP.SVNCHGAGNLLEFAGIRQEQLNRV.P.RVSCS.TRIYTGLT.RYTFKNNG
1ANBSal   SGKI.AEGLV.TGKKFVYLKNNP.SVNCHGAGNLLEFAGIRPEQLNRV.P.RVSCS.SRIYTGLT.RYTFKNNG
1AJBIfl   SGKI.AVKLF.EGKRYIYLKNDG.SAKCRGAGNLLEFAGIRWQQLIRV.PTLHSCNLSRIYSGST.TYTFKNNG
1EWDNnu   SGSI.AAALI.SDKRYVYLKNDG.SPTCRGAGSLLEFSGIRQEQLQRV.PTRHACNFTRTYMGDF.HYTFSNNG
1ABBMma   SGKI.AVGLV.TGKSFIYMKNDG.SAECRGAGSLLEIGGIPPDQQSRV.LMRQSCNSSRIYKGRM.QYTFNNNG
1CNDSme   SGSM.VVGLV.VGKKFVYLRNAG.GKECLGTGNLLEFAGIRPYALDRL.ATPN.CQFQQIYKGKT.QPTFNHNG
1GKDSme   SGSM.VVGLV.VGKKYVYLRNAG.GKECRGTGNLLEFAGIRQDALDRL.SSLN.CQFQQIYMGRT.QPTFNHNG
2AQDAro   SGKM.AIGLV.VGKKYVYLRNYG.GKGCHGTGNLLEFAGIRADAVDRI.PSM..CKFQQVYQGHT.QPNFNYNG
4AADMgu   TGLI.VPGIV.SGKQFAFIRNEG.GTECRGAGGLVEFEGIRAESLADF.PMVHSCKSTRIYSGVT.VYAFTSNG
4ETDMvu   TGQI.VPGIV.SGKQFAFVRNEG.GTECRGAGGLVEFEGIRSDSLANY.PMVHSCPSTRIYSGLT.VYTFVGNG
4KADVag   TGLI.VPGVV.SGKQFAFVRNEG.GTQCRGAGGLVEFEGIRADRLANF.PMVHSCPSTRIYSGVT.VYTFTGNG
4ABDCar   AGLV.IPGSV.SGKQFAFVRNEG.GTNCRGAGGLVEFEGIRVERLEGF.PMVHSCPLTRIYSGLT.VYTFASNG
4DMDGma   AGLV.IPGRV.SGKQFAFVRNEG.GTSCRGAGGLVEFEDIRTERLEGF.PMVHSCPLTRIYSGWT.VYTFASNG
4FUDPvu   AGLV.IPGRV.SGKQFAFVRNEG.GTSCRGAGGLVEFEDIRTERLEGF.PMVHSCPLTRIYSGWT.VYTFASNG
4LJDCca   AGLV.IPGRV.SGKQFAFVRNEG.GTSCRGAGGLVEFEDIRTERLEGF.PMVHSCPSTRIYSGWT.VYTFASNG
4CQDCpa   TGFV.MPGSV.SGKQFAFVRNEG.GTSCRGAGGLVEFEGIRAERLEGF.PMVHSCSSTRIYSGMT.MYTFTNNG
4DNDGra   AGLV.MPGGV.SGKKFAFVRNEG.GTACRGAGGLVEFEGIRPERLESF.PMVHSCSSTRIYSGMT.VYTFTNNG
4GTDTca   AGLV.MPGSV.SGKQFAFVRNEG.GTACRGAGGLVEFEGIRAERLESF.PMVHSCSSTRIYSGMT.VYTFTNNG
4FODPmu   AGLV.SPGTV.SGKQFAFVRNEG.GTSCRGAGGLVEFEGIRAERLEKF.PMVHSCPSTRIYSGLT.VYTFTSNG
4EMDMes   AGMI.IPGIV.SGKQFAFVRNEG.GTSCRGAGGLVEFEGIRAERLENL.HMVHSCPTTRIYSGKT.VYTFSSNG
4FXDRco   TGLI.IPGIV.SGKQFAFVRNEG.GTSCRGAGGLVEFEGIRAERLENF.PMVHSCPTTRIYSGRT.VYTFTSNG
4LRDObi   SGLI.VPGIV.SGKQFAFVRNEG.GTACRGAGGLVEFEGIRSERLENF.PMVHSCPTTRLYSGRT.VYTFVSNG
4LGDJcu   AGQI.VPGVV.SGKQFAFVRNEG.GTSCRGAGGLVEFEGIRAERLENF.PMVHSCPTTRIYSGKT.VYTFANNG
4FPDPtr   AGLV.VPGIV.SGKQFAFVRNEG.GTSCRGAGGLVEFQGIRAERLENL.PMAHSCSTTRIYSGMT.VYTFTTNG
4BLDAne   AGLV.MPGFV.SGKHFAFVRNEG.GTACRGAGGLVEFEGIRPERLEGL.PMVHSCPSTRIYSGMT.VYTFNSNG
4CEDCcl   AGVV.MPGIV.SGKQFAFVRNEG.GTACRGAGGLVEFEGIRPERLEGF.PMVHSCPSTRIYTGMT.MYTFTTNG
4CVDCsi   AGVV.MPGIV.SGKQFAFVRNEG.GTACRGAGGLVEFEGIRPERLEGF.PMVHSCPSTRIYTGMT.MYTFTTNG
4FZDRra   AGLV.MPGIV.SGKQFAFVRNEG.GTSCRGAGGLVEFEGIRPERLEGF.PMVHSCPSTRIYSGMT.VDTFTSNG
4CUDCsa   AGLV.VPGIV.SGKQFAFVRNEG.GTACRGAGGLVEFEGVRPERLENS.PMVHSCPSTRIYSGMT.VYTFSNNG
4DJDFve   AGLV.NPGIV.SGKQFAFVRNEG.GTACRGAGGLVEFEGVRPQRLESL.PMVHSCPSTRIYTGLT.VYTFTSNG
4DHDFre   AGRV.VADIV.SGKQFVFVRNEG.GTACRGAGGLVEFEGVRPERLERF.PMVHSCPSTRIYSGMT.MYTFSNNG
5DBDEgr   SGLI.MPGSV.SGKQFAFVRNEG.GTECRGAGGLVEFEGMRKDRLESF.PMIHSCPTTRIYTGVT.VYTFGSNG
```

```
4DVDIlo AGLI.VPGIV.SGKHFAFVRNEG.GTQCRGAGGLVEFEGIREERLASF.PMIHSCPSIRIYSGTT.VYTFASNG
4KBDIpu AGLI.VPGIV.SGKHFAFVRNEG.GTECRGAGGLVEFEGIREERLASF.PMIHSCSSIRIYSGTT.VYTFASNG
4EVDNbe AGLV.NPGIV.SGKQFAFVRNEG.GTECRGAGGLVEFEGIREERLAIF.PMVHSCPSTRIYSGTT.VYTFTSNG
4GODStu AGHV.NPGMA.SGKQFAFVRNEG.GTECRGAGGLVEFEGIREERLAIL.PMVHFCPSTRIYSGRT.MYTFTSNG
4DZDLch AGLI.TPGHV.SGKQFAFVRNEG.GTACRGAGGLVEFEGIREERLASF.PMVHSCPSTRIYSGVT.VYSFTSNG
4FJDPgr AGLI.MPGSV.SGKQFVFVRNEG.GTSCRGAGGLVEFEGIRRSRLESF.PMVHSCSSTRIYSGMA.VYTFGGNG
4ECDLle AGLI.VPGIV.SGRKFVFVRNEG.GTSCRGAGGLVEFEGIRAERLESF.SMVHSCPTTRIYSGVT.VYTFTSNG
4BIDAly AGLV.MPGSV.SGKQFAFVRNEG.GTDCRGAGGLVEFEGIRAERLERL.PMVHSCPATRIYSGMT.MYTFSANG
4BPDAth AGLV.MPGSV.SGKQFAFVRNEG.GTDCRGAGGLVEFEGIRAERLERL.PMVHSCPATRIYSGMT.MYTFSANG
4CTDCru AGLV.MPGSV.SGKQFAFVRNEG.GTDCRGAGGLVEFEGIRAERLERF.PMVHSCPATRIYSGMT.MYTFSANG
4BYDBra AGLV.MPGSV.SGKQFAFVRNEG.GTDCRGAGGLVEFEDIRAERLERF.PMVHSCPATRIYSGMT.MYTFYANG
4GWDTpa AGLV.MPGSV.SGKQFAFVRNEG.GTDCRGAGGLVEFEDIRAERLERF.PMVHSCPATRIYSGMT.MYTFSANG
4GUDTha AGLV.MPGSV.SGKQFAFVRNEG.GTDCRGAGGLVEFEDIRAERLERF.PMVHSCPATRIYSGLA.MYTFTANG
5BIDAly AGLV.MPGSV.SGKQFAFVRNEG.GTDCRGAGGLVEFEGIRAERLEHF.PMVHSCPKTRIYSGMT.MYMFSGNG
5BPDAth AGLV.MPGSV.SGKQFAFVRNEG.GTDCRGAGGLVEFEGIRAERLEHF.PMVHSCPKTRIYSGMT.MYMFSSNG
5BYDBra AGKV.MPGSV.SGKQFAFVRNEG.GTDCRGAGGLVEFEGIRAERLEHF.PMVHSCPETRIYTGLA.MYTFDGNG
5GWDTpa AGRV.MPGSV.SGKQFAFVRNEG.GTDCRGAGGLVEFEGIRAERLEHF.PMVHSCPETRIYSGLT.MYTFSGNG
5CTDCru AGRV.MPGSV.SGKQFSFVRNEG.GTDCRGAGGLVEFEGIRAERLEHF.PMVHSCPRTRIYSGMT.MYTFSRNG
5GUDTha TGLV.MPGSV.SGKQFAFVRNEG.GTDCRGAGGLVEFEGIRAERLEHF.PRFHSCPATRIYSGMT.MYTFSGNG
4AVDPip AWLI.SPGIV.SGKQFAFVRNEG.GTACRGAGGLVEFEGIRVDRLANF.PMVHSCPTTRIYTGIT.VYTFTSNG
4BCDAan SGLV.LPGNV.SGKQFAFVRNEG.GTACRGAGGLVEFEGIRKDRLANF.PMVHSCPSTRIYSGLT.VYTFPRNG
4DTDHpu SGLV.LPGNV.SGKQFAFVRNEG.GTACRGAGGLVEFEGIRQDRLANF.PMVHSCPSTRIYSGTT.VYTFPRNG
4DDDEsy VGLV.MPGSV.SGKQFAFVRNEG.GTACRGAGGLVEFQGIRNERLENF.PMVHSCPSTRIYLGQT.VYTFTSNG
4KGDBse AGLI.IAGTV.SGKQFAFVRNEG.GTACRGAGGLVEFQGIRTERLAGS.NMLHSCPSTRIYRGMT.VYTFANNG
4ATDPbr ADLI.TPGLV.SGKQFAFVRNEG.GTSCRGAGGLTEFEGIRSERLISL.PTLHSCPSIRIYTGLT.VYSFVSNG
4BJDAme AGLI.TPGLI.SGKQFAFVRNEG.GTSCRGAGGLAEFEGIRAERLANL.PTLHSCPSIRIYTGLT.VYSFVSNG
4DBDEgr SGLI.TPGSI.SGKQFAFIRYEG.GTDCRGAGGLVEFEGIRPSRLESY.PMAHSCKTTRIYTGKT.VYTFASNG
4KEDCca AGLV.SPGPV.SGKQFAFVRNEG.GTACRFAGGLVEFEGIRKERLEGF.LNYHSCPSTRIYTGLT.MYNYESNG
4HKMEgu TGLI.VPGIV.SGKHFAFLRNEG.GNICPGAGALFEFESIRPDRLANF.SSVHSCPSTRIYTGTT.VYSFTSNG
4HLMChe AGLI.VPGIV.SGKQFAFLRNEA.GNICPGAGVLFEFKDIRPERLANF.PLVHSCPATRIYTGTT.VYTFGRNG
4HQMAta AGLV.PEGIV.SGKEFVFLRNEA.GNICPGAGLLFEFFGIRPERLTGFTPAVRMCPVTRIYTGTT.VYTFSSNG
4ITMTur AGLV.PEGIV.SGKEFVFLRNEA.GNICPGAGLLFEFFGIRPERLAGFTPAVRMCPVTRIYMGTT.VYSFSSNG
4HSMBdi AELV.PGGIA.SGKQFAFLRNEA.GNICPGAGVLFEFFGIRPERLAEF.PAVHLCPSTRIYTGTM.DYTFSKNG
4IHMOsa AGLV.PGGIV.SGKQFAFLRNEA.GNICPGAGVLFEFFGIRPERLAEF.PAVHLCPSTRIYTGTT.VYTFTNNG
4ILMPhe AGLV.PGGIV.SGKRFAFLRNEA.GNICPGAGVLFEFFGIRPERLAEF.QAVQLCPSTRIYTGTT.VYTFNNNG
4IOMSit AGLV.PGGIV.SGKQFAFLRNEA.GNICPGAGVLFEFFGIRPERLAAF.PNVHLCPSTRIYTGTT.VYTFGSNG
4INMSbi TGLI.PGGIV.SGKQFAFLRNEA.GNICPGAGVLFEFFGIRPERLAAF.PTVHLCPSTRIYVGTM.DYKFQSNG
4IXMZma TGLI.PGGIV.SGKQFAFLRNEA.GNICPGAGVLFEFFGIRPERLAAF.PTVHLCPSTRIYTGTT.VYSFDKNG
5IOMSit AGLI.TGGIV.SGKQFAFLRNEA.GNICPGAGVLFEFLDIRPERLAQF.PAVHSCASTRIYTGMT.VYTFNQNG
5IXMZma AGLI.TGGMV.SGKQFAFLRNEA.GNICPGAGVLFEFFDIRPERLAQF.PAVHSCASTRIYTGMT.VYTFNQSG
4HWMHdi AGLI.TAGIV.SGKQFAFLRNEA.GNICPGAGTLFEFQGIRPERLASF.PLVNSCPSTRIYTGFT.VYSYQNNG
4IJMPcl AGLI.TAGIV.SGKQFAFLRNEA.GNICPGAGTLFEFQGIRPERLASL.PSVNSCPSTRIYTGTT.VYSYQNNG
4LPMPst AGLI.APGAV.SGKQFAFLRNIG.GTSCAGSGVIFEFSGIRPERLATF.PLVHACSTTRIYLGTA.VYTFASNG
6AADMgu IGAKALTGIL.SGNTLVFVRNVG..NSCRGVGGLLEFAGIRPERLLQV.PSLRSCDFTRMYSGPV.LSLFTRYQ
6AYDTum LGAKALSGIL.SGNTLVFVRNVG..NSCRGVGGLLEFSGIRPERLLQV.PSLRSCDFTRLYSGPV.LSLFTRYQ
6NDDAbr QGAKALSGIL.SGNTLVFVRNVG..NSCRGVGGLLEFSGIRPERLLQV.PSLRTCDFTRLYSGPV.LSLFTRYQ
6ASDMfr QGAKALTGIL.SGNTLVFVRNVG..NSCRGVGGLLEFAGIRSDRLLQV.PSLKSCDFTRMYSGPV.LSLFTRYQ
6ABDCar QGAKSLFGIL.SGNTLVFVRNIG..NSCKGVGGLLEFYGIRSERLSQV.PTLRTCDFTRLYSGPV.LSLFTKYQ
6DMDGma QGAKSLFGIL.SGNTLVFVRNVG..NSCKGVGGLLEFSGIRPERLLQV.PTLRTCDFTRLYSGPV.LSLFTKYQ
6FUDPvu QGAKSLFGIL.SGNTLVFVRNVG..NSCKGVGGLLEFSGIRPERLLQV.PTLRTCDFTRLYSGPV.LSLFTKYQ
6OUDGpo LGAKSLFGIL.SGNTLVFVRNVG..NSCKGVGGLLEFSGIRPERLLQV.PTLRTCDFTRLYSGAV.LSLFTKYQ
6EBDLja IGAKSLFGIL.SGNTLVFVRNVG..NSCKGVGGLLEFSGIRPERLLQV.PTLRTCDFTRLYSGPV.LSLFTKYQ
6ENDMla QGAKALTGIL.SGNTMVFVRNVG..NSCKGVGGLLEFSGIRPERLLQV.PTLRTCDFTRLYSGAV.LSLFTRYQ
6LJDCca LGAKSLYGIL.SENTLVFVRNVG..NSCKGVGGLLEFFGIRPERLLQV.PTLRTCDFTRLYSGPV.LSMFTKYQ
6BCDAan LGAKALSGIL.SGNTMVFVRNVG..NSCRGVGGLLEFSGIRPERLLQV.PTLRSCDFRRLYSGPV.LSLFTQYQ
6BDDAch LGAKALGGIL.SGNTLVFVRNVG..NSCKGVGGLLEFAGIRPERLLQV.PTLKTCDFTRMYSGPV.LSLFTQYQ
6FKDPgr LGAKALSGIL.SGNTLVFVRNVG..NSCKGVGGLLEFAGIRPERLLQV.PTLRTCDFTRMYSGPV.LSLFTQYQ
6NBDVod LGAKALSGIL.SGNTLVFVRNVG..NSCKGVGGLLEFSGIRPERLLQV.PTLKTCDFTRMYSGPV.LSLFTQYQ
6OQDRja LGAKALSGIL.SGNTMMFVRNVG..NSCKGVGGLLEFAGIRPERLLQV.PTLRTCDFTRMYSGPV.LSLFTQYQ
6BLDAne LGAKALSGIL.FGNTLVFVRNVG..NSCKGVGGLLEFAGIRPERLLQI.PTLKSCDFTRMYSGAV.LSLFTQYQ
6BSDBbe LGAKSLSGIL.SGNTLVFVRNVG..NSCKGVGGLLEFAGIRPERLLQV.PTLKSCDFTRMYSGAV.LSLFTQYQ
6DZDLch LGAKSLSGIL.SGNTLVFVRNVG..NSCKGVGGLLEFAGIRPERLLQV.PSLKSCDFTRMYSGAV.LSLFTQYQ
6GTDTca LGAKSLSGIL.AGNTLVFVRNVG..NSCKGVGGLLEFAGIRPERLLQI.PNLKSCDFTRMYSGAV.LSLFTQYQ
6CQDCpa LGAKSLSGIL.SDNTLVFVRNVG..NSCKGVGGLLEFAGIRPERLLQI.PTLKSCDFTRMYSGAV.LSLLTQYQ
6DNDGra LGAKSLSGIL.SGNTLVFVRNVG..NSCKGVGGLLEFAGIRPERLLQI.SNLKSCDFTRMYSGAV.LSLFTQYQ
6FPDPtr LGAKALSGIL.SGNTLVFVRNVG..NSCKGVGGLLEFAGIKAERLLQV.PTLKTCDFTRLYSGAV.LSLFTQYQ
6CJDCic LGAKALGGIP.SGNTLVFVRNVG..NSCKGVGGLLEFAGIRPERLLQV.PTLKSCDFTRMYSGPV.LSLFTQYQ
6OPDHpe LGAKSFSGIL.SGNTLVFVRNVG..NSCQGVGGLLEFSGIRPERLLQV.PTLKTCDFTRMYSGPV.LSMFTQYQ
6CLDCla LGAKSLNGIL.SGNTLVFVRNVG..NSCKGVGGLLEFAGIRPERLQQE.PTLKTCDFTRLYSGPV.LSLFTKYQ
6OYDCsa LGAKSLNGIL.SGNTLVFVRNVG..NSCKGVGGLLEFAGIRPERLQQE.PTLKTCDFTRLYSGPV.LSLFTKYQ
6CODCme LGAKSLNGIL.SGNTLVFVRNVG..NSCKGVGGLLEFSGIRPERLQQE.PTLKTCDFTRLYSGPV.LSLFTKYQ
6DJDFve LGAESLSGIL.SGNTLVFVRNVG..NSCKGVGGLLEFAGIRPERLLQD.PTLKTCDFTRLYSGPV.LSLFTKYQ
6FYDRpa LGAKSLSGIL.SGNTLVFVRNVG..NSCKGVGGLLEFAGIRPERLLQD.PTLKTCDFTRLYSGPV.LSLFTKYQ
6FQDPpe LGAKSLSGIL.SGNTLVFVRNIG..NSCKGVGGLLEFAGIRPERLQQD.PTLKTCDFTRLYSGAV.LSLFTKYQ
6ELDMdo IGAKALSGIL.SGNTLVFVRNVG..NSCKGVGGLLEFAGIRPERLQQD.PTLRTCDFARLYSGPV.LSLFTKYQ
7CYDSda LGEKALTGIL.SGNTLVFVRNVG..NSCKGVGGLLEFAGIRPERLLQV.PNLKTCDFTRMYSGAV.LSLFTQYQ
6LRDObi LGDKALIGIL.SGNTLVFVRNVG..NSCKGVGGLLEFSGIRPERLLQV.PTLRSCDFTRMYSGAI.LSLFTRYQ
6AWDAre IGAKALNGIL.SGNTLIFVRNVG..N.CRGVGGLLEFAGIRPERLLQV.PLLKSCDFTRLYSGPV.LSLFTRYQ
6ECDLle LGSKALTAIP.SGSTIVFVRNVG..NSCKGVGGLLEFAGIRPERLLQV.PTLRSCDFTRLYSGPV.LSLFTQYQ
6EIDLus LGSKALTAIP.SGSTIVFVRNVG..NSCKGVGGLLEFAGIRPERLLLV.PTLRSCDFTRLYSGPV.LSLFTQYQ
6CEDCcl LGAKPLGGFL.SSNTLVFVRNVG..NSCKGVGGLLEFAGIRPERLLQI.PTLKSCDFARMYSGPV.LSLFTQYQ
6CVDCsi LGAKPLGGFL.SSNTLVFVRNVG..NSCKGVGGLLEFAGIRPERLLQI.PTLKSCDFARMYSGPV.LSLFTQYQ
6EVDNbe QGAKALSGIL.SGNTLVFVRNVG..NSCRGVGGLLEFYGIHPERLLQV.PSLKSCDFTRLYSGPV.LSAFTRYQ
6GJDSly QGAKALSGIL.SGNTLVFVRNVG..NSCRGVGGLLEFYGIHPERLLQV.PSLKSCDFTRLYSGPV.LSAFTRYQ
6GODStu QGAKALSGIL.SGNTLVFVRNVG..NSCRGVGGLLEFYGIHPERLLQV.PSLKSCDFTRLYSGPV.LSAFTRYQ
6BIDAly PGSKALSGLL.SGNTMAFVRNVG..NSCKGVGGLVEFSGIRPERLLQI.PSLKSCDFTRMYSGPI.LSLFTRYQ
6BPDAth PGSKALSGLL.SGNTMAFVRNVG..NSCKGVGGLVEFSGIRPERLLQI.PSLKSCDFTRMYSGPI.LSLFTRYQ
6CTDCru PGSKALSGLL.SGNTMAFVRNVG..NSCRGVGGLVEFSGIRPERLLQI.PSLKSCDFTRMYSGPI.LSLFTRYQ
6GUDTha PGSKALSGLL.SGNTMAFVRNVG..NSCKGVGGLVEFAGIRPERLLQI.PSLKSCDFTRMYSGPI.LSLFTRYQ
6GWDTpa PGSKALSGLL.SGNTMAFVRNVG..NSCKGVGGLVEFSGIRPERLLQI.PSLKSCDFTRMYSGPI.LSLFTRYQ
6BYDBra PGSKALSGLL.SGNTMAFVRNVG..NSCKGVGGLVEFSGIRPERLLQI.PSLKSCDFTRMYSGPI.LSLFTRYQ
6GMDSsa PGSKALTGIL.SGNTMVFVRNVG..NSCKGVGGLLEFSGIRQERLLQI.PNLKSCDFTRMYSGAI.LSLFTRYQ
6CUDCsa QGAKALSGIL.SGNTLVFVRNVG..SSCRGAGGLLEFAGIRPDRLYQV.PTLKTCQFTRMYSGAV.LSMFTQYQ
6DHDFre LGAKALTGIL.AGNSLVFVRNVG..NSCRGAGGLLEFAGIRPDRLLQV.PSLKSCQFTRLYSGAV.LSMFTQYQ
6OTDWin LGAKALSGIL.MGNTLVFLRNVG..NSCKGVGGLLEFSGIRPERLLQV.PTLKSCDLTRMYSGPV.LSLFTQYQ
6CFDCcr LGAKAFSGYL.SDNTLVFVRNVG..NSCKGVGGLLEFSGIRPERLQQI.PTLKTCDFTRFYSGPV.LSRFTKYQ
6ACBAtr PNEKPLSGIL.SGNTLAFVRNVG..NSCKGVGGLLEFAGIRPERLLQV.PTLKTCDFTRLYSGAA.LNDWTHYQ
6AIBIau LGAKALNGIL.SGNTLAFVRNVG..NSCKGVGGLLEFAGIRPERLLQV.PTLKSCDFTRLYSGAA.LSDFSHYQ
6AKBLse LGDKALNGIL.SGNTLAFVRNVG..NACKGVGGLLEFAGIRPERLLQV.PTLKTCDFTRLYSGAA.LSDFSHYQ
6HKMEgu LGAKALSGIL.SGNTLAFVRNVG..NSCKGVGGLLEFAGIRPERLLQV.PTLKTCDFTRLYSGAA.LSDWTHYQ
6IKMPda LGAKALSGIL.SGNTLAFVRNVG..NSCKGVGGLLEFAGIRPERLLQV.PTLKTCDFTRLYSGAA.LSDWTHYQ
6HWMHdi LGAKALSGIL.LGNTLAFVRNVG..NLCKGVGGLLEFAGIRPERLLQI.PTLKSCDFTRLYSGAA.LRDWTHYK
6APDAco LGAKTFTGIL.SGNTLAFVRNVG..NSCKGVGGLLEFSGIRPQRLLEV.PSLKTCDFTRMYSGAV.LSVWAHYQ
```

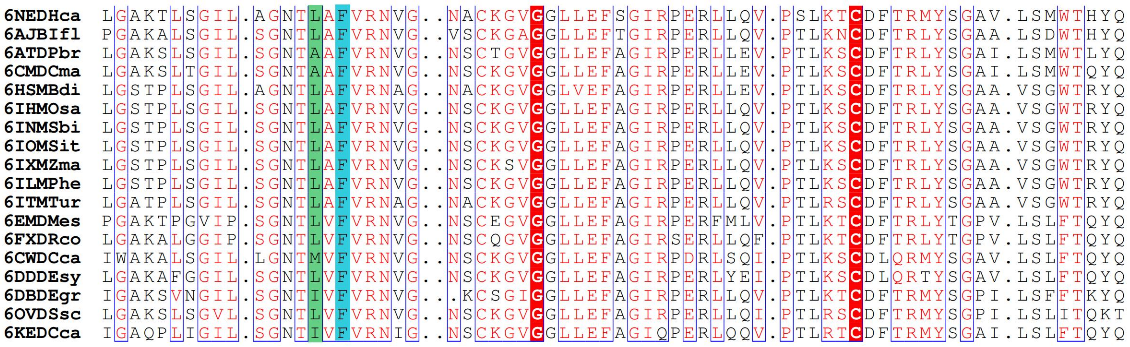

Supplementary Figure S3: Protein alignment of IDs of BR receptors from different species. conserved residues are indicated in red. Residue 597 and Residue 599 are highlighted with green and bule, respectively.

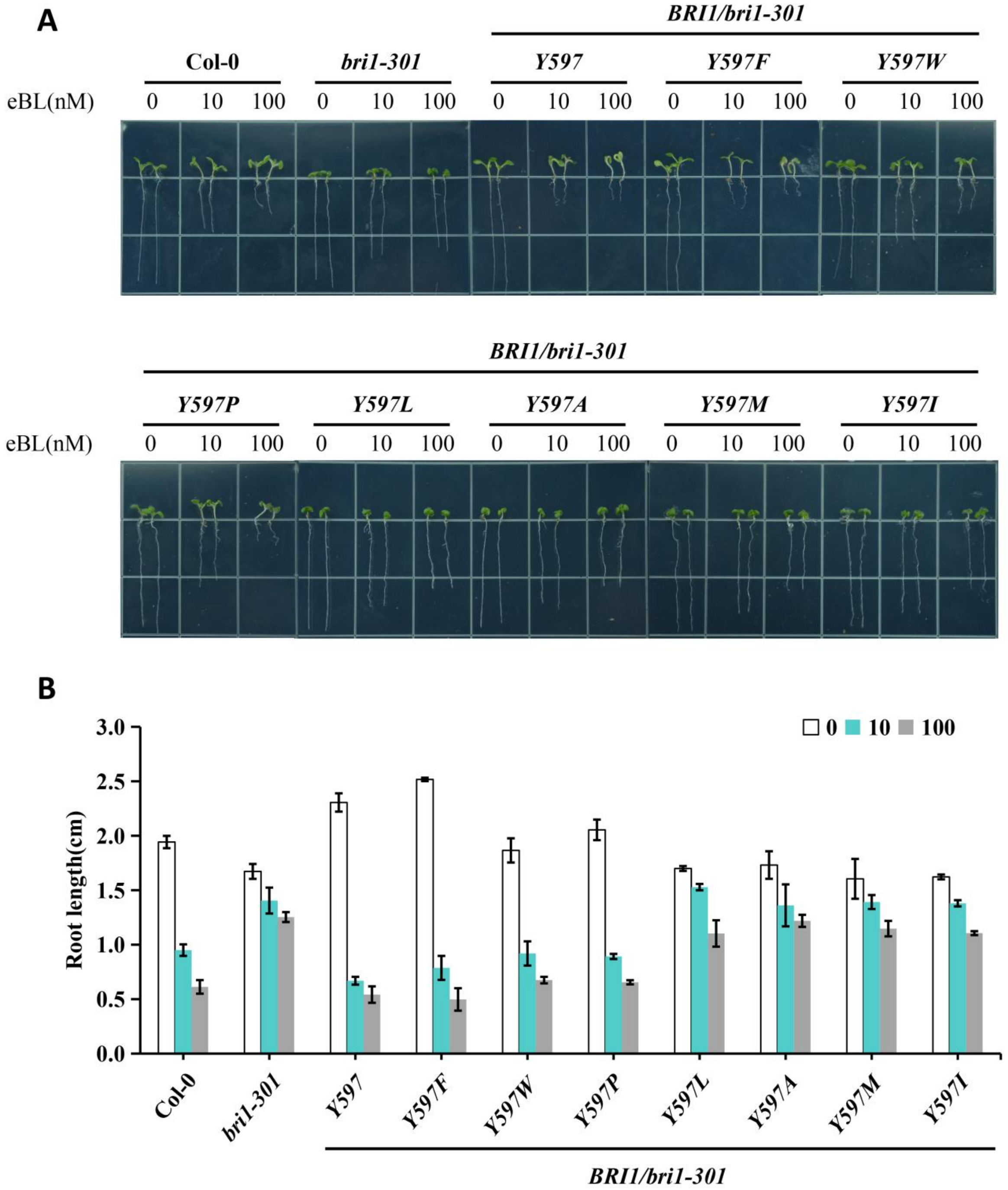

Supplementary Figure S4. eBL sensitivity analysis for residue 597 of AtBRI1. **(A)** Roots of transgenic plants expressing AtBRI1 mutated at the residue of 597 treated with eBL. **(B)** The column representations of root lengths of eBL-treated transgenic plants shown in **(A)**. The results were represented as means±SD (n=10). Different colours represent different concentrations of eBL. All the experiments were repeated independently three times with similar results.

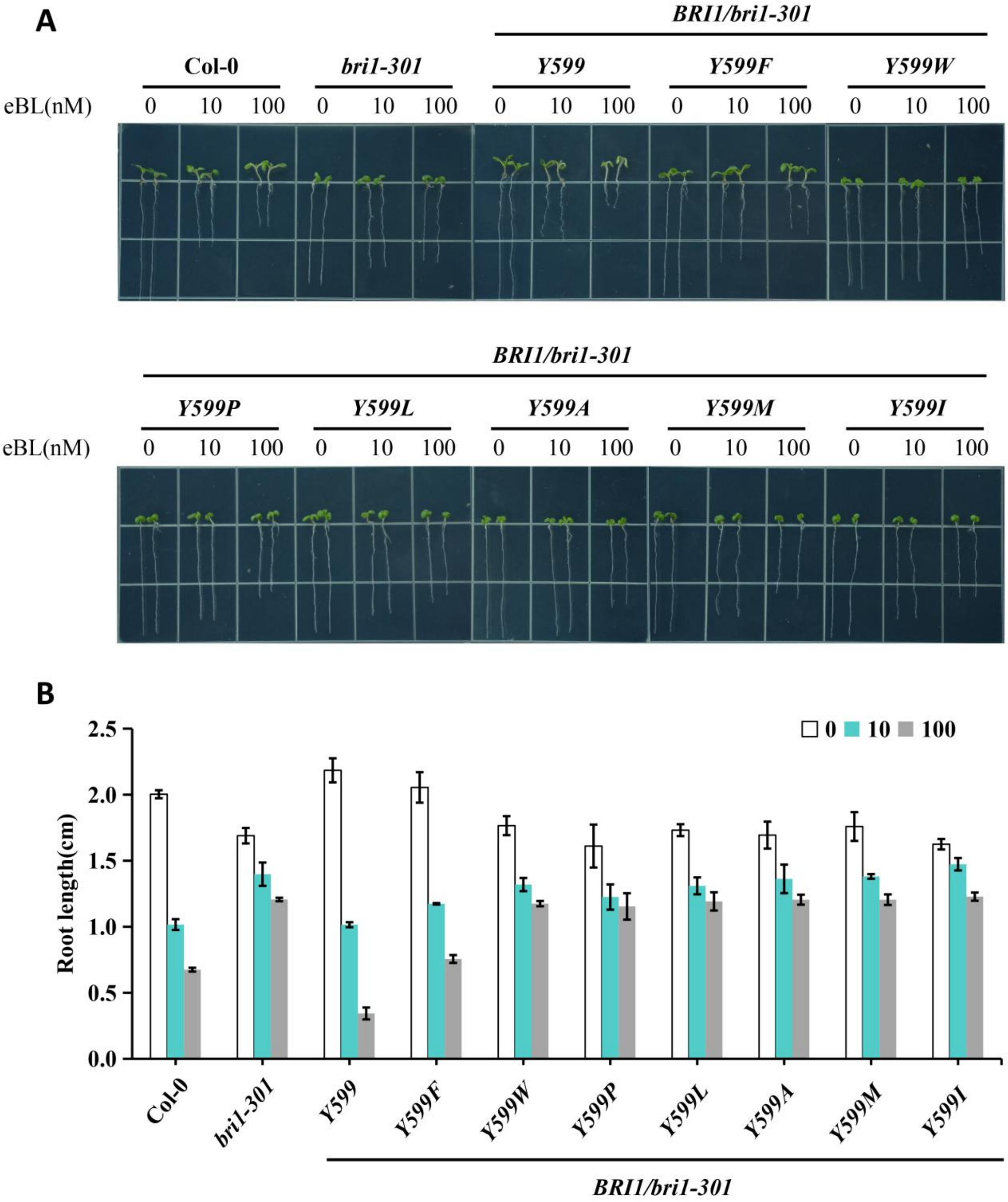

Supplementary Figure S5. eBL sensitivity analysis for residue 599 of AtBRI1. **(A)** Roots of transgenic plants expressing AtBRI1 mutated at the residue of 599 treated with eBL. **(B)** The column representations of root lengths of eBL-treated transgenic plants shown in **(A)**. The results were represented as means±SD (n=10). Different colours represent different concentrations of eBL. All the experiments were repeated independently three times with similar results.

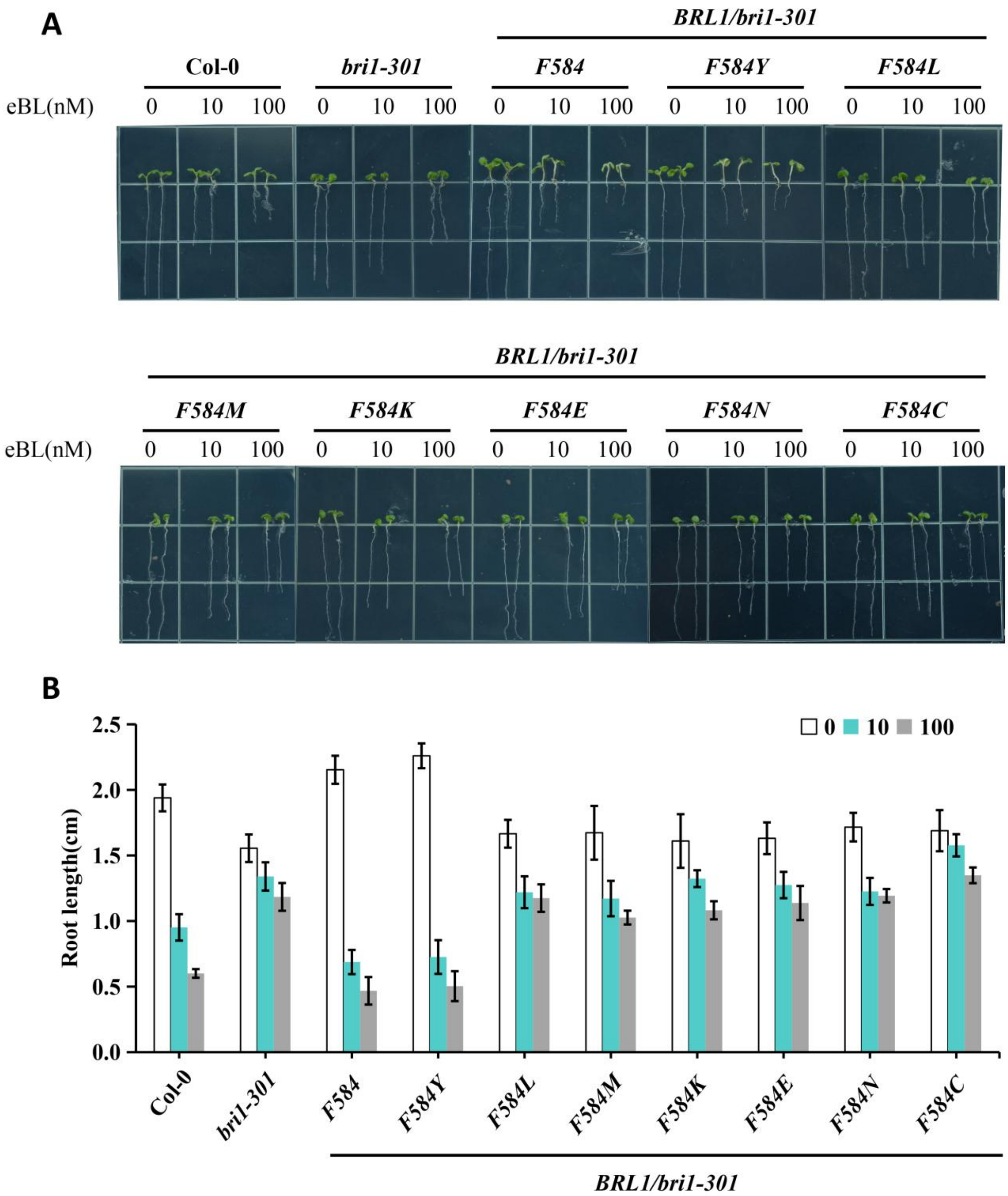

Supplementary Figure S6. eBL sensitivity analysis for residue 584 of AtBRL1(corresponding to the residue 597 of AtBRI1). **(A)** Roots of transgenic plants expressing AtBRL1 mutated in the residue of 584 treated with eBL. **(B)** The column representations of root lengths of eBL-treated transgenic plants shown in **(A)**. The results were represented as means±SD (n=10). Different colours represent different concentrations of eBL. All the experiments were repeated independently three times with similar results.

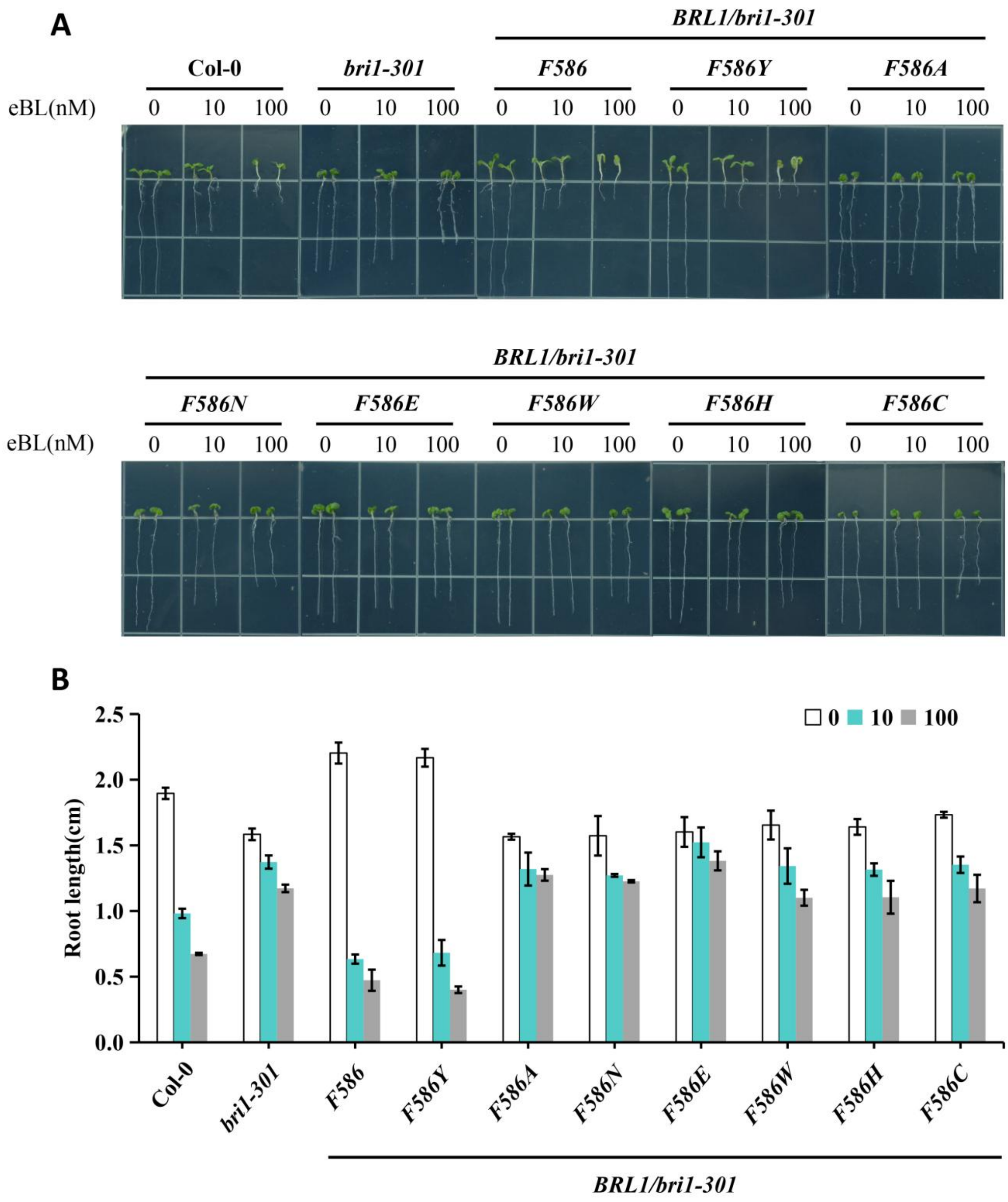

Supplementary Figure S7. eBL sensitivity analysis for residue 586 of AtBRL1(corresponding to the residue 599 of AtBRI1). **(A)** Roots of transgenic plants expressing AtBRL1 mutated in the residue of 586 treated with eBL. **(B)** The column representations of root lengths of eBL-treated transgenic plants shown in **(A)**. The results were represented as means±SD (n=10). Different colours represent different concentrations of eBL. All the experiments were repeated independently three times with similar results.

Supplementary Table S2. The primer used in this study.

Used for gene cloning	
AtBRI1-F-Kpn1	TGCGGTACCATGAAGACTTTTTCAAGCTTCTTTC
AtBRI1-R-Sal1	GCGGTCGACTAATTTTCCTTCAGGAACTTCTT
AtBRI1Y597F-F	CATCGCCGGTAAGAGGTTTGTTTATC
AtBRI1Y597F-R	TTTGATAAACAAACCTCTTACCGGCGATG
AtBRI1Y597L-F	CATCGCCGGTAAGAGGCTCGTTTATC
AtBRI1Y597L-R	TGATAAACGAGCCTCTTACCGGCGATG
AtBRI1Y597I-F	CATCGCCGGTAAGAGGATCGTTTATCA
AtBRI1Y597I-R	TTGATAAACGATCCTCTTACCGGCGATG
AtBRI1Y597M-F	CATCGCCGGTAAGAGGATGGTTTATC
AtBRI1Y597M-R	TTTGATAAACCATCCTCTTACCGGCGAT
AtBRI1Y597V-F	CATCGCCGGTAAGAGGGTCGTTTATC
AtBRI1Y597V-R	TTTGATAAACGACCCTCTTACCGGCGATG
AtBRI1Y597S-F	CATCGCCGGTAAGAGGTCCGTTTATC
AtBRI1Y597S-R	TTGATAAACGGACCTCTTACCGGCGATG
AtBRI1Y597P-F	CATCGCCGGTAAGAGGCCCGTTTATC
AtBRI1Y597P-R	TTTGATAAACGGGCCTCTTACCGGCGATG
AtBRI1Y597T-F	CATCGCCGGTAAGAGGACCGTTTATC
AtBRI1Y597T-R	TTTGATAAACGGTCCTCTTACCGGCGATG
AtBRI1Y597A-F	CATCGCCGGTAAGAGGGCCGTTTATC
AtBRI1Y597A-R	TTTGATAAACGGCCCTCTTACCGGCGATG
AtBRI1Y597H-F	CATCGCCGGTAAGAGGCACGTTTATC
AtBRI1Y597H-R	TTTGATAAACGTGCCTCTTACCGGCGATG
AtBRI1Y597Q-F	CATCGCCGGTAAGAGGCAGGTTTATC
AtBRI1Y597Q-R	TTTGATAAACCTGCCTCTTACCGGCGATG
AtBRI1Y597N-F	CATCGCCGGTAAGAGGAACGTTTATC
AtBRI1Y597N-R	TTTGATAAACGTTCCTCTTACCGGCGATG
AtBRI1Y597K-F	CATCGCCGGTAAGAGGAAGGTTTATC
AtBRI1Y597K-R	TTTGATAAACCTTCCTCTTACCGGCGATG
AtBRI1Y597D-F	CATCGCCGGTAAGAGGGACGTTTATC
AtBRI1Y597D-R	TTTGATAAACGTCCCTCTTACCGGCGATG
AtBRI1Y597E-F	CATCGCCGGTAAGAGGGAGGTTTATC
AtBRI1Y597E-R	TTTGATAAACCTCCCTCTTACCGGCGATG
AtBRI1Y597C-F	TCGCCGGTAAGAGGTGCGTTTATCAAAAAC
AtBRI1Y597C-R	GTTTTTGATAAACGCACCTCTTACCGGCGAT
AtBRI1Y597W-F	ATCGCCGGTAAGAGGTGGGTTTATCAAAAAC
AtBRI1Y597W-R	GTTTTTGATAAACCCACCTCTTACCGGCGAT
AtBRI1Y597R-F	CATCGCCGGTAAGAGGCGCGTTTATC
AtBRI1Y597R-R	TTTGATAAACGCGCCTCTTACCGGCGATG
AtBRI1Y597G-F	CATCGCCGGTAAGAGGGGCGTTTATC
AtBRI1Y597G-R	TTTGATAAACGCCCCTCTTACCGGCGATG
AtBRI1Y599F-F	GGTAAGAGGTACGTTTTTATCAAAAACGATGGGAT
AtBRI1Y599F-R	TCATCCCATCGTTTTTGATAAAAACGTACCTCTTACC
AtBRI1Y599L-F	GGTAAGAGGTACGTTCTAATCAAAAACGATGGGAT
AtBRI1Y599L-R	TCATCCCATCGTTTTTGATTAGAACGTACCTCTTACC
AtBRI1Y599I-F	CGGTAAGAGGTACGTTATTATCAAAAACGATGGGATG
AtBRI1Y599I-R	CCCATCGTTTTTGATAATAACGTACCTCTTACC
AtBRI1Y599M-F	GGTAAGAGGTACGTTATGATCAAAAACGATGGGAT
AtBRI1Y599M-R	CCCATCGTTTTTGATCATAACGTACCTCTTAC
AtBRI1Y599V-F	GGTAAGAGGTACGTTGTTATCAAAAACGATGGGAT
AtBRI1Y599V-R	CCCATCGTTTTTGATAACAACGTACCTCTTAC
AtBRI1Y599S-F	GGTAAGAGGTACGTTTCTATCAAAAACGATGGGATG

AtBRI1Y599S-R	CCCATCGTTTTTGATAGAAACGTACCTCTTAC
AtBRI1Y599P-F	GGTAAGAGGTACGTTCCTATCAAAAACGATG
AtBRI1Y599P-R	CCCATCGTTTTTGATAGGAACGTACCTCTTAC
AtBRI1Y599T-F	GGTAAGAGGTACGTTACTATCAAAAACGATGGGAT
AtBRI1Y599T-R	CCCATCGTTTTTGATAGTAACGTACCTCTTAC
AtBRI1Y599A-F	GGTAAGAGGTACGTTGCTATCAAAAACGATGGGAT
AtBRI1Y599A-R	CCCATCGTTTTTGATAGCAACGTACCTCTTAC
AtBRI1Y599H-F	GGTAAGAGGTACGTTCATCAAAAACGATGGGAT
AtBRI1Y599H-R	CCCATCGTTTTTGATGAACGTACCTCTTAC
AtBRI1Y599Q-F	GGTAAGAGGTACGTTCAAATCAAAAACGATGGGAT
AtBRI1Y599Q-R	CCCATCGTTTTTGATTTGAACGTACCTCTTAC
AtBRI1Y599N-F	CGGTAAGAGGTACGTTAATCAAAAACGATGGGAT
AtBRI1Y599N-R	CCCATCGTTTTTGATTAACGTACCTCTTACC
AtBRI1Y599K-F	CCGGTAAGAGGTACGTTAAAATCAAAAACGATGGGAT
AtBRI1Y599K-R	CCCATCGTTTTTGATTTTAACGTACCTCTTACC
AtBRI1Y599D-F	GGTAAGAGGTACGTTGATCAAAAACGATGGGAT
AtBRI1Y599D-R	CCCATCGTTTTTGATCAACGTACCTCTTAC
AtBRI1Y599E-F	GGTAAGAGGTACGTTGAAATCAAAAACGATGGGAT
AtBRI1Y599E-R	CCCATCGTTTTTGATTTCAACGTACCTCTTAC
AtBRI1Y599C-F	GGTAAGAGGTACGTTTGTATCAAAAACGATGGGAT
AtBRI1Y599C-R	CCCATCGTTTTTGATACAAACGTACCTCTTAC
AtBRI1Y599W-F	GGTAAGAGGTACGTTTGGATCAAAAACGATGGGAT
AtBRI1Y599W-R	CCCATCGTTTTTGATCCAAACGTACCTCTTAC
AtBRI1Y599R-F	GGTAAGAGGTACGTTCGTATCAAAAACGATG
AtBRI1Y599R-R	CCCATCGTTTTTGATACGAACGTACCTCTTACC
AtBRI1Y599G-F	GGTAAGAGGTACGTTGGTATCAAAAACGATGGGAT
AtBRI1Y599G-R	CCCATCGTTTTTGATACCAACGTACCTCTTAC
AtBRL1- F-Kpn1	GGGGTACCATGAAGCAGAGATGGCTGTTAGTGT
AtBRL1- R-Sal1	GCGTCGACTTAAGGCTCCTTATCTCGCGATTCT
AtBRL1F584Y-F	AGGTAAACAGTATGCGTTTGTGAG
AtBRL1F584Y-R	TCACAAACGCATACTGTTTACCTG
AtBRL1F584L-F	AGGTAAACAGTTAGCGTTTGTGAG
AtBRL1F584L-R	TCACAAACGCTAACTGTTTACCTG
AtBRL1F584M-F	CAGGTAAACAGATGGCGTTTGTGAG
AtBRL1F584M-R	CTCACAAACGCCATCTGTTTACCTG
AtBRL1F584H-F	CAGGTAAACAGCATGCGTTTGTGAG
AtBRL1F584H-R	CTCACAAACGCATGCTGTTTACCTG
AtBRL1F584N-F	CAGGTAAACAGAATGCGTTTGTGAG
AtBRL1F584N-R	TCTCACAAACGCATTCTGTTTACCT
AtBRL1F584K-F	CAGGTAAACAGAAAGCGTTTGTGAG
AtBRL1F584K-R	TCTCACAAACGCTTTCTGTTTACCT
AtBRL1F584E-F	CAGGTAAACAGGAAGCGTTTGTGAG
AtBRL1F584E-R	CTCACAAACGCTTCCTGTTTACCTG
AtBRL1F584C-F	CAGGTAAACAGTGTGCGTTTGTGAG
AtBRL1F584C-R	CTCACAAACGCACACTGTTTACCTG
AtBRL1F586Y-F	GGTAAACAGTTTGCGTATGTGAGAAAC
AtBRL1F586Y-R	GTTTCTCACATACGCAAACTGTTTACC
AtBRL1F586S-F	GGTAAACAGTTTGCGTCTGTGAGAAAC
AtBRL1F586S-R	GTTTCTCACAGACGCAAACTGTTTACC
AtBRL1F586P-F	ACAGTTTGCGCCTGTGAGAAACGAAGGT
AtBRL1F586P-R	GTTTCTCACAGGCGCAAACTGTTTACCT
AtBRL1F586T-F	TAAACAGTTTGCGACTGTGAGAAACGAAG
AtBRL1F586T-R	TCGTTTCTCACAGTCGCAAACTGTTTACCT
AtBRL1F586A-F	AACAGTTTGCGGCTGTGAGAAACGAAGGT
AtBRL1F586A-R	CGTTTCTCACAGCCGCAAACTGTTTACCTG

AtBRL1F586H-F	AAACAGTTTGCGCATGTGAGAAACGAAGG
AtBRL1F586H-R	TTCTCACATGCGCAAACTGTTTACCTG
AtBRL1F586N-F	TTAAGGCTCCTTATCTCGCGATTCT
AtBRL1F586N-R	TTCTCACATTCGCAAACTGTTTACC
AtBRL1F586K-F	AACAGTTTGCGAAAGTGAGAAACGAAG
AtBRL1F586K-R	TTCGTTTCTCACTTTCGCAAACTGTTTAC
AtBRL1F586E-F	AAACAGTTTGCGGAAGTGAGAAACGAAGG
AtBRL1F586E-R	TCTCACTTCCGCAAACTGTTTACCTG
AtBRL1F586C-F	ATGAAGCAGAGATGGCTGTTAGTGT
AtBRL1F586C-R	TTCGTTTCTCACACACGCAAACTGTTTAC
AtBRL1F586W-F	GGTAAACAGTTTGCGTGGGTGAG
AtBRL1F586W-R	TTCTCACCCACGCAAACTGTTTACC
